# Dietary management and growth outcomes in children with propionic acidemia: A natural history study

**DOI:** 10.1002/jmd2.12234

**Published:** 2021-06-14

**Authors:** Haneen Saleemani, Csilla Egri, Gabriella Horvath, Sylvia Stockler‐Ipsiroglu, Rajavel Elango

**Affiliations:** ^1^ BC Children's Hospital Research Institute, BC Children's Hospital Vancouver British Columbia Canada; ^2^ Faculty of Land and Food Systems University of British Columbia Vancouver British Columbia Canada; ^3^ Faculty of Applied Medical Sciences, Department of Clinical Nutrition King Abdulaziz University Jeddah Saudi Arabia; ^4^ Division of Biochemical Genetics BC Children's Hospital Vancouver British Columbia Canada; ^5^ Department of Pediatrics University of British Columbia Vancouver British Columbia Canada; ^6^ School of Population and Public Health, University of British Columbia Vancouver British Columbia Canada

**Keywords:** branched‐chain amino acids, growth, medical formula, propionic acidemia, protein

## Abstract

**Background:**

Propionic acidemia (PROP) is an autosomal recessive inherited deficiency of propionyl‐CoA carboxylase (PCC) which is involved in the catalytic breakdown of the amino acids valine, isoleucine, methionine, and threonine. PROP nutritional management is based on dietary protein restriction and use of special medical formulas which are free of the offending amino acids, but are enriched in leucine. The resulting imbalance among branched‐chain amino acids negatively impacts plasma concentrations of valine and isoleucine, which might impact growth in children with PROP.

**Objective and Methods:**

Our primary objective was to describe dietary protein and calorie intake and their impact on long‐term growth outcomes of four PROP patients. This was accomplished through a longitudinal retrospective chart review following the cohort from birth to 18 years.

**Results:**

All children (n = 4) had poor growth outcomes with persistently reduced height‐for‐age *Z* scores, and elevated weight and body mass index (BMI) *Z* scores. Energy intakes for all subjects were within 80% to 120% of the dietary reference intakes for age. All children had low intakes of intact protein compared with current guidelines and were supplemented with medical formula and single l‐amino acids (valine and/or isoleucine), which led to the excess consumption of total protein.

**Conclusion:**

Despite adequate total protein and energy intakes, all children had persistently low height *Z* scores. Restricted intact protein consumption together with the abundant use of medical formula could have affected overall growth. To optimize dietary management in patients with PROP, further research is needed to determine the optimal intake of medical formula relative to intact protein.

**Abbreviations**
BCAAs: branched‐chain amino acids
BMI: body mass index
DRIs: dietary reference intakes
ILE: isoleucine
LEU: leucine
MET: methionine
PCC: propionyl‐CoA carboxylase
PROP: propionic acidemia
RDA: recommended dietary allowance
THR: threonine
VAL: valine

**Synopsis**

In a longitudinal retrospective chart review, children with propionic acidemia show inadequate growth indicated by reduced height *Z* scores, elevated weight, and BMI *Z* scores, likely influenced by reduced intake of intact protein:energy ratio.

## 1 INTRODUCTION

Propionic acidemia (PROP) (OMIM 606054) is an autosomal recessive, inherited metabolic disorder caused by a defect in the mitochondrial enzyme propionyl‐coenzyme A (CoA) carboxylase (PCC) (EC 6.4.1.3). PCC catalyzes the reversible biotin‐dependent conversion of propionyl‐CoA to D‐methylmalonyl‐CoA. Propionyl CoA is an intermediate in the catabolism of isoleucine (ILE) and valine (VAL), two of the three branched‐chain amino acids (BCAAs), as well as threonine (THR), methionine (MET), and odd chain fatty acids.^1^ PROP is considered an ultra‐rare disorder, with similar rates across all regions estimated to be 1 in 100 000, except for regions in the Middle East and North Africa, where most inherited metabolic disorders have higher incidents.^2^

Nutritional management is a mainstay in the treatment of PROP with the goal to reduce the accumulation of toxic metabolites by restricting dietary protein sources of the propiogenic amino acids (ILE, VAL, MET, and THR)^3^ and to prevent endogenous protein catabolism, by providing sufficient energy to meet metabolic demands.^4^ Given the paucity of nutrition‐based studies in PROP, dietary management is based on individualized clinical and laboratory assessments^5^ and general recommendations adhering to dietary reference intakes (DRIs) for energy and protein intake.^3^, ^6^ In addition to consuming a diet restricted in intact protein, subjects with PROP are usually supplemented with special medical formulas, that is formulated to contain no propiogenic precursors (ILE, VAL, MET, and THR) and normal to high amounts of other amino acids to ensure sufficient protein intake for optimal growth.^7^ Although there is limited evidence on efficacy studies that support the use of medical formula in PROP, a European survey stated that about 81% of 47 centers across Europe prescribe medical formula regularly with majority of centers prescribing medical formula that provide more than 50% of total protein.^8^ The role of medical formula use in PROP is still questionable with arguments against their use, due to their formulation of imbalanced BCAA content.^7^, ^9^

The primary objective of this study is to describe historical and current dietary therapeutic practices and their impacts on long‐term growth outcomes in four PROP patients through a natural history study. A retrospective chart review was conducted on the four PROP patients between 1999 and 2018. We hypothesize that the patients would have poor growth outcomes associated with high medical formula consumption relative to intact protein consumption.

## 2 METHODS

### 2.1 Subjects

This study was approved by the University of British Columbia Children's and Women's Research Ethics Board (H19‐02912). A retrospective chart review was conducted on four pediatric patients with PROP followed in the Biochemical Diseases Clinic at BC Children's Hospital, Vancouver, British Columbia, Canada. Patients were two sibling pairs, with the older siblings (PROP‐01, PROP‐03) diagnosed after an acute metabolic decompensation in the neonatal period, and the younger siblings diagnosed right after birth as they were screened (Table 1). Longitudinal data were collected on dietary intake and growth outcomes between 1999 and 2018, following the cohort from age 0 to 18 years. Data were extracted from medical and dietetic clinic records, at times when patients were metabolically stable.

**TABLE 1:** Patient characteristics

Patient ID	Age at diagnosis	Sex	Number of sick days^a^	Mutation
PROP‐01	4 wk	F	Birth‐1 y (45 d) 1‐3 y (22 d) 4‐8 y (165 d)	PCCA^b^ Homozygous c.134_135delTA p. Leu45TyrX
PROP‐02	2 wk	M	Birth‐1 y (12 d) 1‐3 y (42 d) 4‐8 y (28 d)	PCCA Homozygous c.134_135delTA p. Leu45TyrX
PROP‐03	6 mo.	F	Birth‐1 y (43 d) 1‐3 y (13 d) 4‐8 y (47 d)	PCCB^c^ Homozygous c.337C>T p. Arg113X
PROP‐04	Prenatal	F	Birth‐1 y (39 d) 1‐3 y (63 d) 4‐7 y (26 d)	PCCB Homozygous c.337C>T p. Arg113X

^a^ Calculated as number of days during the analysis period; Sick day formula was used to supply 120% EER for age, with 0% to 50% intact protein during this time.

^b^ Propionyl Co‐A carboxylase alpha subunit.

^c^ Propionyl Co‐A carboxylase beta subunit.

### 2.2 Dietary management

Following diagnostic confirmation of PROP and/or stabilization after the initial metabolic crisis, all patients were started on nutrition therapy with restricted natural protein intake and additional medical formula depleted of the offending amino acids (VAL, ILE, THR, MET). Medical formula was provided under a public health care grant. Additional nutritional supplements, such as calcium, iron, other trace elements, and vitamins, were added according to needs. In addition to the nutrition therapy, all patients received supplementation of l‐carnitine (100‐300 mg/kg) orally. Metronidazole to halt growth of gut bacteria producing propionic acid were given in alternating intervals 2 weeks on, 2 weeks off, as well as various supplements were also given as needed. Low intact protein convenience foods were at the expense of the families until 2014, and then provided under a public health care grant. Intact protein restriction was based around the age‐related DRI and fine‐tuned according to biomarkers indicating metabolic stability, nutritional and growth‐related needs. The following guidelines were used to inform protein and energy supply: prior to 2004, the Ross recommendations were used as dietary management guidelines,^10^ from 2004 to 2007, dietary management was based on the Sass recommendations.^11^ A comparison among these guidelines for total protein intake is presented in Figure S1.

Metabolic stability was determined via regular monitoring of blood gas, anion gap, plasma ammonia levels, and urine ketones. Other biomarkers such as blood lactate levels and urine citrate/methylcitrate ratio were also determined.^12^ Nutritional and growth‐related needs were determined via regular monitoring of growth parameters (weight, height, body mass index [BMI]), plasma concentrations of amino acids, albumin and prealbumin, calcium, and other trace minerals and vitamins. Adjustments of nutrition therapy prescriptions were made by the metabolic dietitian and communicated to the patient's caregivers via phone or email. Patients were seen in the metabolic clinic at 1‐ to 3‐month intervals within the first year of life and in 4‐ to 6‐month intervals thereafter. Clinic visits included assessments with the dietitian, the clinic nurse and the physician and occasionally with a psychologist and/or a social worker.

During sick times, patients received a home sick day formula which supplied 120% of estimated energy requirement (EER) and 50% to 100% of the regularly prescribed intact protein, while the missing amount of intact protein was replaced by respective amounts of medical PROP formula. Zero percent intact protein was prescribed when admitted with acute metabolic decompensation, advancing gradually to 50% intact protein intake, then 100% upon achievement of metabolic stability.

### 2.3 Data collection

#### 2.3.1 Anthropometric data

Anthropometric data were collected from medical and dietetic records during clinic visits. Weight and length for children under 2 years of age were obtained by standard techniques using digital baby weighing scales and crown‐heel length on a scale length board, respectively. Weight and height for children older than 2 years of age were measured using digital scale and a stadiometer, respectively. Measurements were performed by the dietitian or clinic nurse. BMI was calculated using the equation kg/m^2^. Anthropometric measurements were expressed as age‐ and gender‐specific *Z*‐scores, using the WHO Anthro and Anthroplus software for 0 to 5 years of age and 5 to 19 years of age, respectively. For 0 to 5 years, indicators included: weight for age, weight for height, and height for age. For 5 to 19 years, indicators included: weight for age, height for age, and BMI for age (WHO AnthroPlus for personal computers Manual: Software for assessing growth of the world's children and adolescents. Geneva: WHO, 2009) (http://www.who.int/growthref/tools/en/).

#### 2.3.2 Dietary data

Dietary data were collected on the basis of formula recipes delivered via tube feeding and/or food records for the oral intake. Dietary intake was analyzed manually following Ross protocols (1999‐2004) and then using the MetabolicPro software from Genetic Metabolic Dietitian International (GMDI). Dietary data were only collected when patients were metabolically stable. We excluded data during sick days as patients were consuming a special sick day formula, number of sick days for each subject was calculated, and is presented with patient characteristics (Table 1). Formula composition was obtained from the respective manufacturers. Dietary data represent reported, rather than prescribed intake. Protein intake (g/kg/d) as total protein was calculated by adding intact protein, protein from medical formula, and single amino acid (l‐isoleucine and l‐valine) supplements. Protein intake was also separately calculated as g/kg/d of intact protein and protein from medical formula. Energy intake was collected as kcal/kg/d. The protein to energy (P:E) ratio was calculated based on amount of total protein in grams per 100 kcal per day. The calculated P:E ratio values were compared with P:E values associated with optimal growth in patients with inborn error of protein metabolism described by Evans et al (>1.5 to <2.9 g/100 kcal/day).^13^ Since patients were nutritionally managed using different guidelines at different times, actual intakes were compared with the 2001 Ross recommendation,^10^ the 2004 Sass recommendation,^11^ and with the most recent 2019 guidelines for PROP from Genetic Metabolic Dietitian International (GMDI).^3^ For comparison purposes, protein and energy intakes were combined for all four subjects according to age groups (0‐6 months, 7‐12 months, 1‐3 years, 4‐8 years, 9‐13 years, and 14‐18 years).

### 2.4 Statistical analysis

Statistical analysis was performed using GraphPad Prism 8.0 (GraphPad Software Inc, California). Descriptive statistics were used to compare actual energy and protein intakes with different recommendation; all data were expressed as median and range (minimum‐maximum). Growth *Z* scores were reported according to age groups: for 0 to 5 years (weight for height, weight for age, and height for age), for 5 to 10 years (height for age, weight for age, and BMI for age), for 10 to 18 years (height for age, and BMI for age). Growth data were also expressed as median and range (minimum‐maximum).

## 3 RESULTS

### 3.1 Patient characteristics

Three female and one male patient with PROP were followed in the Biochemical Disease Clinic at BC Children's Hospital (Table 1). All patients had gastrostomy tubes receiving part or their entire daily nutritional needs through bolus or continuous feeds. One patient (PROP‐03) received growth hormone therapy starting at age 9 years for documented growth hormone deficiency. All patients had relatively good metabolic control and had hospital admissions only with moderate hyperammonemic episodes. Despite having no major metabolic crises, one patient (PROP‐04) died of cardiomyopathy at 10 years of age.

### 3.2 Growth data

**FIGURE 1:**
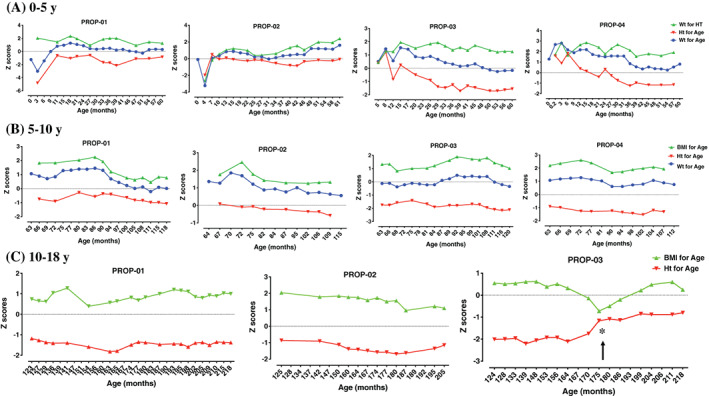
Growth data *Z* scores (sex‐specific charts) in children with propionic acidemia (PROP). A, Weight for height, height for age, and weight for age from 0 to 5 years old. B, BMI, height, and weight for age from 5 to 10 years old. C, BMI and height for age from 10 to 18 years old. *Growth hormone therapy started

All four children had poor growth outcomes with persistently reduced height *Z* scores, and elevated weight and BMI *Z* scores (Figure 1). During the first 5 years of life, all patients had a median *Z* score of 1.6 (range: 1.04‐2) for weight for height, and −0.717 (range: −1.36 to −0.2) for height. From 5 to 10 years, height *Z* scores declined to a median of −1.03 (range: −1.78 to −0.23) for all patients. However, their median BMI for age *Z* scores were at 1.35 with a range of 1.01 to 2.03 that translates to a BMI percentile of (>84 and <95) and classifies them as overweight. Between 10 and 18 years, height *Z* scores, calculated on three patients, decreased even more to a median of −1.4. After 10 years of age, the WHO recommends not using weight for age, as it does not distinguish between height and BMI during pubertal growth spurts. Thus, our data are only presented as BMI for age from 10 to 18 years. PROP‐03, who had received growth hormone replacement therapy at age 9, had an improvement in height and BMI *Z* scores (indicated by an arrow in Figure 1C).

The reported parental heights for PROP‐01/‐02 father's height is 175 cm and mother's height is 162 cm; for PROP‐03/‐04, father's height is 177 cm and mother's height is 161 cm. Based on midparental height compared to patient's actual heights an estimation as shown earlier by Wright and Cheetham,^14^ we observed the following: midparenteral height for PROP‐01 is 174.5 cm (0 *Z* score) vs actual height at age 18 years was 166.5 cm (−1.4 *Z* score); midparenteral height for PROP‐02 is 161.5 cm (0 *Z* score) vs actual height at age 18 years was 153 cm (−1.4 *Z* score). For PROP‐03, midparenteral height is 162 cm (0 *Z* score) vs actual height age 18 years was 157.8 cm (−1.8 *Z* score). PROP‐04 passed away at age 10 years, and so we were not able to calculate these values.

We also collected data (in February 2021) from healthy siblings of PROP‐01/‐02: male 16 years (height = 177.8 cm, weight = 81.65 kg) and male 10 years (height = 149.9 cm, weight = 54.4 kg). We observed that their WHO height‐for‐age *Z* scores showed that they were both ~0 to +1 *Z* scores (Figure S2).

### 3.3 Dietary data

**FIGURE 2:**
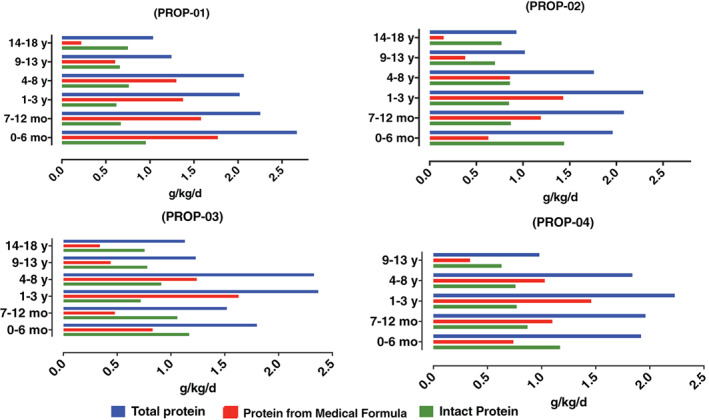
Protein intake in children with propionic acidemia (PROP)

Protein intakes, including total, intact, and protein from medical formula in g/kg/d, are presented for each patient in Figure 2. Energy intakes for all subjects were within 80% to 120% of the DRI for age (Figure S3).^6^ A comparison among the different guidelines for total protein intake suggests no major differences between the 2019 GMDI^3^ and 2004 Sass et al^11^ guidelines (Figure S1). However, the Ross recommendations^11^ for total protein is 92% to 108% higher than GMDI recommended intakes, and 59% to 50% higher than Sass recommendations for 0 to 6 months and 7 to 12 months, respectively. This indicates that compared to the most recent guidelines, the Ross recommendations were significantly higher in total protein intakes.

Median percentages of intact protein vs protein from medical formula varied for each patient and for each age group (Figure 2).

The protein to energy ratio (P:E) in g/100 kcal/d was calculated for both intact vs total protein and presented for all subjects in Table 2. Protein to energy ratio of (>1.5 to <2.9 g/100 kcal) was found by Evans et al,^13^ to be associated with optimal growth outcomes, for all pediatric age ranges. In our results, median total P:E ratio for 1 to 3 years was 2.75/100 kcal, and within the optimal ratio. However, the median intact P:E ratio for the same age was 0.9 g/100 kcal and well below the reference optimal ratio.

**TABLE 2:** Dietary protein to energy ratio (grams of protein/100 kcal/d)

Age	Actual intakes^a^		Recommended protein to energy ratio^b^ ^13^
	Total protein:energy	Intact protein:energy	
**PROP‐01** 0‐6 mo.	2.75	0.98	>1.5 to <2.9
7‐12 mo.	2.47	0.73	
1‐3 y	2.66	0.81	
4‐8 y	3.09	1.13	
9‐13 y	2.7	1.4	
14‐18 y	2.99	2.1	
**PROP‐02** 0‐6 mo.	2.06	1.5	>1.5 to <2.9
7‐12 mo.	2.21	0.92	
1‐3 y	2.55	0.94	
4‐8 y	3.09	1.5	
9‐13 y	2.55	1.7	
14‐18 y	2.66	2.2	
**PROP‐03** 0‐6 mo.	1.89	1.23	>1.5 to <2.9
7‐12 mo.	1.68	1.17	
1‐3 y	2.85	0.86	
4‐8 y	4.2	1.66	
9‐13 y	1.61	1.65	
14‐18 y	2.97	1.98	
**PROP‐04** 0‐6 mo.	2.06	1.26	>1.5 to <2.9
7‐12 mo.	2.77	1.23	
1‐3 y	3	1.03	
4‐8 y	2.23	1.33	
9‐13 y	2.06	1.29	

^a^ Actual intakes are reported as medians.

^b^ Protein to energy ratio associated with optimal growth in subjects with inborn error of metabolism.

## 4 DISCUSSION

In this retrospective chart review study, we show growth data in association with nutrition therapy of four patients with PROP followed longitudinally from birth to the 18 years at the same center between 1999 and 2018. We observed that all patients had poor growth outcomes, with persistently reduced height‐for‐age *Z* scores, but at the same time had elevated weight and BMI for age *Z* scores. Poor growth outcomes with respect to height rather than weight in subjects with PROP have been well described,^13^, ^15^, ^16^, ^17^, ^18^, ^19^ although in majority of the reports, most patients also had poor weight gain. In the largest case series comprising 55 patients with PROP^19^ observed an early onset and progressive growth restriction when compared to the patients' target heights calculated from parental heights; however, the majority of patients still had a height higher than −2SD. While no correlation with protein intake could be established in that study, IGF‐1 concentrations, which can serve as a biomarker for protein malnutrition, were decreased in the majority of patients.^19^ Recently, Molema et al in a longitudinal nationwide retrospective analysis of Methylmalonic Acidemia (MMA) and PROP patients also showed total protein intake, and especially additional amino acid intake was associated with impairments in growth and cognitive impairment, and raises significant concerns about the role of increased protein intake in these patients.^20^

Our center's nutrition therapy protocol was based on restriction of intact protein, supplementation of medical formula (depleted of the offending amino acids VAL, ILE, THR, and MET), and provision of high calorie intake. During the 19‐year observation period, the targets for total protein intake were informed by three consecutively published international guidelines. These guidelines showed considerable discrepancies within the (0‐12 month) age, with the Ross guidelines recommending 3 to 3.5 g protein/kg/d, and the Sass and GMDI guidelines recommending much lower amounts ranging from 1.5 to 2 g protein/kg/d.^3^, ^10^, ^11^ Our patients were all born between 1999 and 2004 when the Ross guidelines^11^ were standard of care and thus received high total protein amounts during their first year of life. Our patients' consumption of intact protein between the age 1 and 3 years was only 70% of recommended dietary allowance (RDA) according to the current GMDI guidelines.^3^ However, total protein intake for the same age was 179% of RDA, in keeping with the Ross guidelines, used as standard of care at the time. Given the inherently limited tolerance of intact protein, the proportion of protein provided by medical formula ended up accounting up to 67% of total protein intake during certain life‐periods of our patients (Figure 2). Thus, while total protein intake in our patients was well above the DRI, the proportion of intact protein tended to be low. The chronic restriction of intact protein likely constitutes an important cause of the observed growth deficiencies. It is also worth mentioning that with the new 2019 guidelines,^3^ all patients' intakes of intact protein increased with age, and accordingly, medical formula consumption decreased.

Imbalanced amino acid intake could be another reason for reduced growth. Medical formula used in PROP is not only depleted of VAL and ILE but also at the same time is enriched in leucine (LEU). Enhanced VAL and ILE oxidation in the presence of abundant LEU, known as BCAA antagonism, makes both VAL and ILE less available for anabolism and thus could contribute to growth deficiency despite high total protein intakes.^9^, ^21^ In a cross‐sectional study, Molema et al^18^, ^22^ reported that subjects receiving medical formula had significantly lower plasma values of VAL and ILE compared to subjects not receiving medical formula. Moreover, plasma VAL was positively associated with the amount of intact protein consumption and negatively associated with the amount of LEU in medical formula used.^18^, ^22^ Another study presenting the long‐term outcomes and dietary data on PROP reported low to very low plasma VAL and ILE in all subjects.^15^ In the current study, we did not report plasma amino acid concentrations, but the fact that subjects needed to be supplemented with single amino acids (VAL and ILE) indicates that their plasma values were deficient. High LEU intakes can negatively impact the other two BCAAs (ILEU and VAL), by suppressing their plasma concentrations below normal ranges,^23^, ^24^ limiting total protein synthesis, and restricting growth.^21^ We are currently performing in vivo stable isotope based experiments in PROP patients and healthy controls to measure protein synthesis to better understand the nutritional effects of an imbalanced BCAA ratio with LEU‐enriched medical formula, similar to our recent proof‐of concept stable isotope based nutritional studies in pyridoxine‐dependent epilepsy.^25^

Children heights are also attributable to parenteral height, and our patients' height data might have been influenced by this.^26^ Our analysis of the PROP patients' attained height in comparison with midparental heights, as described earlier by Wright and Cheetham,^14^ showed that the height *Z* scores were lower compared to the expected height *Z* scores. Despite the reduced height, our patients had elevated weight for height/age *Z* scores during the first 5 years of life, as well as elevated BMI *Z* scores after the age of 5 years. All children had energy intakes within recommendations (80%‐120% of EER) at different ages. It is likely that subjects could have been physically inactive due to concomitant manifestations such as muscular hypotonia, neurological deficits, cardiomyopathy, and general discomfort during physical activity and accordingly would have needed less energy, as shown earlier for MMA by Hauser et al.^27^ In the absence of direct energy expenditure assessments, this could not be determined for our patients. Resting energy expenditure measured by indirect calorimetry in PROP patients earlier was noted to be 20% less than calculated requirements.^4^ Another explanation for the disproportional weight gain in our patients could be the frequent use of sick day formulas with energy at 120% of EER for age, combined with either none or low (0%‐50%) intact protein. On average, all our patients had 32 sick days in the first 6 months of life, and 35 days from 1 to 3 years (Table 1).

Dietary protein and energy are interdependent, and an adequate energy intake ensures efficient protein utilization.^28^ Thus, P:E is useful to assess diets in patients. Evans et al showed that a P:E ratio of >1.5 to <2.9 g of protein/100 kcal is associated with optimal growth in subjects with inborn errors of metabolism including PROP.^13^ In a natural history study on organic acidemia patients, the median intact P:E ratio was 1.23 g/100 kcal/d and it was positively associated with height *Z* score.^18^ In the current study, calculated P:E ratios from total protein showed a median ratio that was within the optimal ratio. However, when intact protein intake was used to calculate the ratio, the results were lower than the optimal ratio (Table 2). Inadequate intact protein in relation to the energy supply might have been another reason for the discordant height vs weight gain in our patients. Future studies could include body composition assessments in PROP subjects and observe changes longitudinally with measurement of P:E ratios.

Limitations associated with this study first and foremost include the small sample size. Although all four patients were treated at the same clinic, there was high variability in their dietary treatments. Therefore, we could not perform any statistical correlations between diet and growth outcomes. While the dietary data represented actual intakes rather than prescribed, it did not describe intakes of the BCAAs. Although the longitudinal growth data over 18 years enabled us to confidently document growth patterns, there was no information on body composition (lean body mass vs fat mass). One of the patients (PROP‐03) was found to be growth hormone deficient, which prompted the clinic to treat with growth hormones. This could have been the reason for the skewed height data in this patient's chart.

## 5 CONCLUSION

Despite adequate amount of total protein and energy intake meeting respective recommendations, growth outcomes in all PROP patients were below standards, especially for height *Z* scores. Although total protein intakes were above recommendations, intact protein intakes were low, with abundant use of medical formula and single amino acid supplements. Optimizing dietary management in PROP, balancing medical formula relative to intact protein, and an assessment of intact P:E ratio is recommended.

## CONFLICT OF INTEREST

The authors declare no potential conflict of interest.

## AUTHOR CONTRIBUTIONS

Haneen Saleemani collected data and participated in study design, data analysis, and data interpretations. Haneen Saleemani drafted the manuscript. Csilla Egri and Gabriella Horvath contributed to data collection and patient care and together with Sylvia Stockler‐Ipsiroglu and Rajavel Elango cosupervised study design, data analysis and interpretation, and edited the manuscript. All authors have read and approved the final version of this paper.

## ETHICS STATEMENT

The study was approved by the University of British Columbia and BC Children's Hospital Research Ethics Board (H19‐02912).

## Supporting information

**SUPPLEMENTARY FIGURE 1** Comparison with Different Guidelines for Total Protein Intakes in patients with propionic Acidemia (PROP)

**SUPPLEMENTARY FIGURE 2** PROP‐01, 02 healthy siblings: Scores as per 2021 @ age of 16y, and 10y

**SUPPLEMENTARY FIGURE 3** Energy Intakes in children with Propionic Acidemia (PROP) compared to 80‐120% Recommended Dietary allowance (RDA) for energy
