# Supplementary material for: Dietary management and growth outcomes in children with propionic acidemia: A natural history study
Source: JIMD Rep. 2021 Jun 14;61(1):67–75. doi: 10.1002/jmd2.12234 (PMC8411103; doi:10.1002/jmd2.12234)
Supplement: Supplementary file 1 — SUPPLEMENTARY FIGURE 1 Comparison with Different Guidelines for Total Protein Intakes in patients with propionic Acidemia (PROP) [file JMD2-61-67-s003.pdf]

SUPPLEMENTARY FIGURE 1. Comparison with Different Guidelines for Total Protein Intakes in patients with propionic Acidemia (PROP)

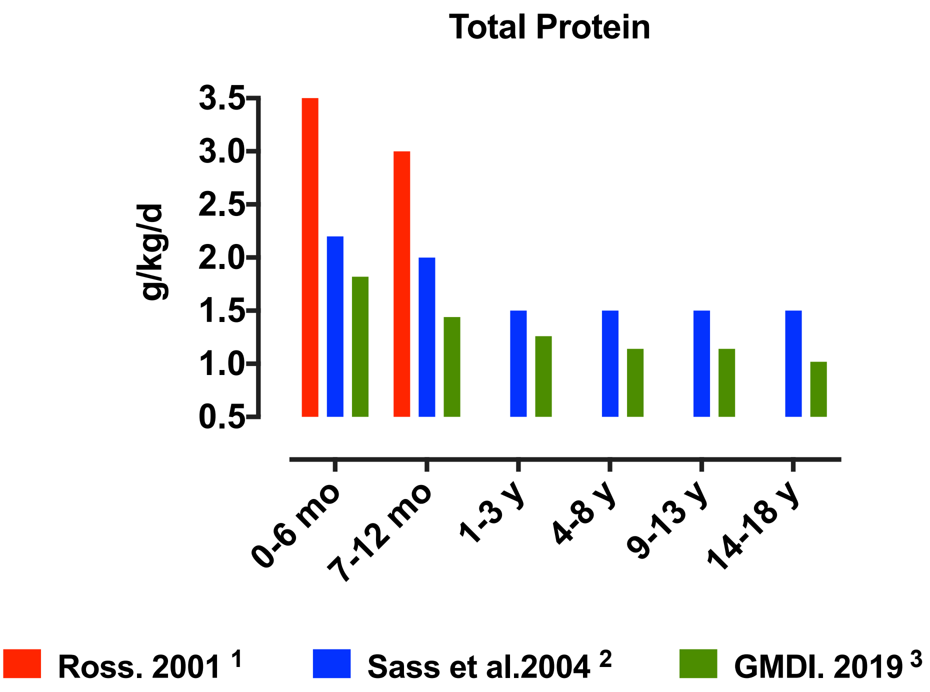

[1](Phyllis B.Acosta and Steven Yannicelli, 2001). Recommendations were reported in g.kg/d only for 0-12 months of age

[2] (Sass et al., 2004)

[3] (Jurecki et al., 2019)
