# Supplementary material for: Dietary management and growth outcomes in children with propionic acidemia: A natural history study
Source: JIMD Rep. 2021 Jun 14;61(1):67–75. doi: 10.1002/jmd2.12234 (PMC8411103; doi:10.1002/jmd2.12234)
Supplement: Supplementary file 2 — SUPPLEMENTARY FIGURE 2 PROP‐01, 02 healthy siblings: Scores as per 2021 @ age of 16y, and 10y [file JMD2-61-67-s001.pdf]

Height-for-age BOYS

Supplementary Figure 2.
PROP-01,02 healthy siblings: Scores as per 2021 @ age of 16y, and 10y

World Health Organization

5 to 19 years (z-scores)

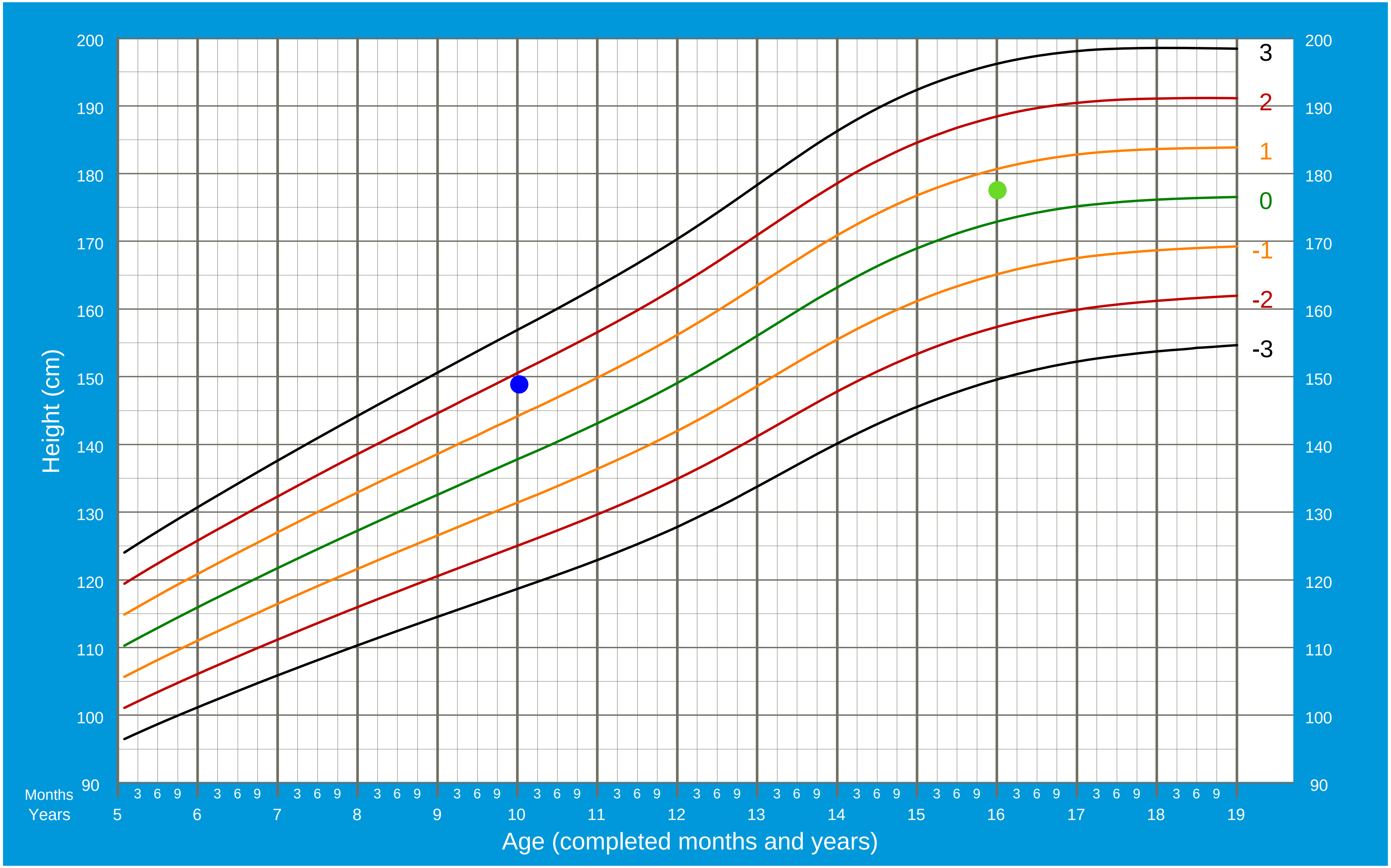

2007 WHO Reference
