# Supplementary material for: Dietary management and growth outcomes in children with propionic acidemia: A natural history study
Source: JIMD Rep. 2021 Jun 14;61(1):67–75. doi: 10.1002/jmd2.12234 (PMC8411103; doi:10.1002/jmd2.12234)
Supplement: Supplementary file 3 — SUPPLEMENTARY FIGURE 3 Energy Intakes in children with Propionic Acidemia (PROP) compared to 80‐120% Recommended Dietary allowance (RDA) for energy [file JMD2-61-67-s002.pdf]

SUPPLEMENTARY FIGURE 3. Energy Intakes in children with Propionic Acidemia (PROP) compared to 80-120% Recommended Dietary allowance (RDA) for energy

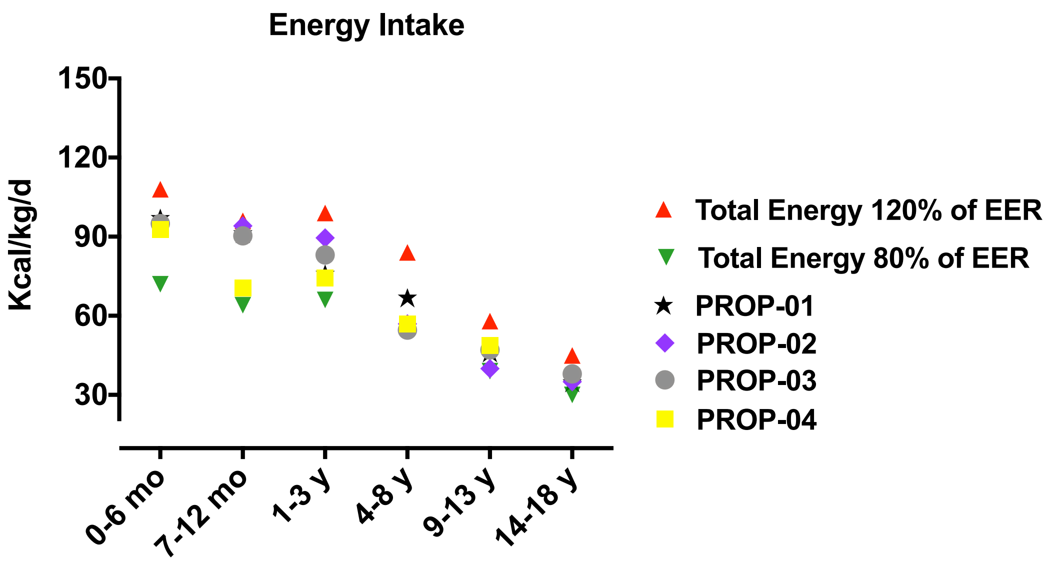

EER, estimated energy requirements
